# Decreases in the Serum VLDL-TG/Non-VLDL-TG Ratio from Early Stages of Chronic Hepatitis C: Alterations in TG-Rich Lipoprotein Levels

**DOI:** 10.1371/journal.pone.0017309

**Published:** 2011-02-25

**Authors:** Motoi Nishimura, Haruna Yamamoto, Toshihiko Yoshida, Masanori Seimiya, Yuji Sawabe, Kazuyuki Matsushita, Hiroshi Umemura, Kazuyuki Sogawa, Hirotaka Takizawa, Osamu Yokosuka, Fumio Nomura

**Affiliations:** 1 Department of Molecular Diagnosis, Graduate School of Medicine, Chiba University, Chiba City, Japan; 2 Division of Laboratory Medicine, Chiba University Hospital, Chiba City, Japan; 3 Clinical Proteomics Research Center, Chiba University Hospital, Chiba City, Japan; 4 Kashiwado Clinic, Kashiwado Memorial Foundation, Chiba City, Japan; 5 Department of Medicine and Clinical Oncology, Graduate School of Medicine, Chiba University, Chiba City, Japan; University of South Carolina School of Medicine, United States of America

## Abstract

**Background:**

The liver secretes very-low-density lipoproteins (VLDLs) and plays a key role in lipid metabolism. Plasma total triglyceride (TG) level variations have been studied in patients with hepatitis C virus (HCV)-related chronic hepatitis (CH-C). However, the results of these studies are variable. A homogenous assay protocol was recently proposed to directly measure the TG content in VLDL (VLDL-TG) and VLDL remnants.

**Methodology/Principal Findings:**

Using the assay protocol, we determined serum VLDL-TG levels in 69 fasting patients with biopsy-proven HCV-related chronic liver disease and 50 healthy subjects. Patients were classified into stages F0–F4 using the 5-point Desmet scale. Serum total TG levels in patients with non-cirrhotic (F1–F3) CH-C did not demonstrate significant differences compared with healthy subjects, but serum VLDL-TG levels did demonstrate significant differences. Mean serum VLDL-TG levels tended to decrease with disease progression from F1 to F4 (cirrhosis). Compared with healthy subjects, serum non-VLDL-TG levels significantly increased in patients with stages F2 and F3 CH-C; however, we observed no significant difference in patients with liver cirrhosis. Furthermore, the serum VLDL-TG/non-VLDL-TG ratio, when taken, demonstrated a significant decrease in patients with CH-C from the mildest stage F1 onward.

**Conclusions/Significance:**

The decrease in serum VLDL-TG levels was attenuated by increase in non-VLDL-TG levels in patients with non-cirrhotic CH-C, resulting in comparable total TG levels. Results of previous studies though variable, were confirmed to have a logical basis. The decrease in the serum VLDL-TG/non-VLDL-TG ratio as early as stage F1 demonstrated TG metabolic alterations in early stages of CH-C for the first time. The involvement of TG metabolism in CH-C pathogenesis has been established in experimental animals, while conventional TG measurements are generally considered as poor indicators of CH-C progression in clinical practice. The serum VLDL-TG/non-VLDL-TG ratio, which focuses on TG metabolic alterations, may be an early indicator of CH-C.

## Introduction

Plasma triglyceride (TG)-rich lipoproteins comprise a mixture of lipoprotein species that are characterized by differences in density and apolipoprotein composition. In the fasting state, plasma TGs are predominantly transported by very-low-density lipoproteins (VLDLs) secreted by the liver [1]. In the fed state, plasma TGs are substantially transported by VLDLs as well as chylomicrons (CMs) secreted by the intestine. Because the liver plays a key role in lipoprotein metabolism, alterations in lipoprotein metabolism in hepatobiliary diseases may be of practical as well as of theoretical interest.

In many countries, chronic hepatitis C virus (HCV) infection is the leading cause of chronic hepatitis, liver cirrhosis, and hepatocellular carcinoma. Interactions between chronic HCV infection and lipid metabolism have been suggested. Some studies indicate a higher prevalence of hypocholesterolemia [2] and hypobetalipoproteinemia [3] in patients infected with HCV than healthy subjects (controls). These changes may be more common among patients infected with HCV genotype 3. These patients develop hepatic steatosis more frequently than those infected with other genotypes [2].

Furthermore, plasma TG level variations have been reported in patients with HCV-related chronic hepatitis (CH-C) [2], [4]. The results of previous studies on plasma TG levels, however, are variable. One report indicates significantly lower total plasma TG levels in patients with CH-C than in non-HCV controls [4]; however, it should be mentioned that patients in this study were not confirmed by liver biopsy and 51% of these patients might have significant fibrosis or cirrhosis [4]. In another report, total TG levels in patients with biopsy-proven CH-C were comparable to those in control subjects [2]. Hence, in CH-C clinical practices, plasma TG level is not considered as a sensitive laboratory test. However, experiments with animals and cultured cells have established relationships between TG metabolism, HCV infection, and CH-C. TG metabolism is not only affected by HCV infection [5], [6], but it also exerts an influence on HCV replication [7].

Because TGs in plasma are transported by a mixture of different lipoprotein species, such as CMs, VLDLs, and VLDL remnants [also called intermediate-density lipoproteins (IDLs)], selective measurement of these TG-rich lipoproteins is essential to obtain further insight into TG metabolic alterations under pathological conditions.

Compared to low-density lipoprotein (LDL) and high-density lipoprotein (HDL), technological difficulties have hampered specific measurement of VLDL although TGs are transported mainly by VLDL in the fasting state; these conditions differ from those surrounding cholesterols. Cholesterols are transported mainly by LDL [1], and specific measurement of cholesterol content in LDL is a commonly-used laboratory test in clinical practice.

To overcome these difficulties, Okada et al. [8] proposed a homogenous assay protocol to directly measure the TG content in VLDL and VLDL remnants. In the present study, we refer to this measurement as VLDL-TG, conventional TG measurement as total TG, and total TG minus VLDL-TG as non-VLDL-TG; thus non-VLDL-TG includes TG content in CM (CM-TG), TG content in LDL (LDL-TG) and TG content in HDL (HDL-TG). We utilized this new method and evaluated these serum TG profiles in biopsy-proven CH-C patients.

## Methods

### Ethics Statement

The study was approved by the Human Ethics Committee of Chiba University. Written informed consent was obtained from all the subjects. The Human Ethics Committee of Chiba University also approved the use of residual sera collected during routine laboratory sampling from the patients for the study, provided that strict anonymity was maintained. The patient sera were irrevocably anonymized and unlinked from the persons who provided the sample.

### Objectives

As mentioned in the Introduction section, it remains to be established whether or not the serum total TG levels in patients with CH-C are significantly lower than those in healthy subjects. However, this fact appears unreasonable from the viewpoints that the liver is a central organ for lipid metabolism and that total cholesterol, which is a representative lipid in blood, is evidently lower in patients with CH-C than in healthy subjects [2], [4]. There must be a rational explanation for this discrepancy. Thus, we considered elucidating the explanations for this by investigating the serum TG profile using the VLDL-TG method developed by Okada et al. [8].

### Participants

This study included 69 patients with untreated HCV-related chronic liver disease who had been hospitalized for liver biopsy at the Chiba University Hospital. The control group consisted of 50 apparently healthy, fasting subjects who had sought an annual medical check-up at the Kashiwado Clinic. All subjects were negative for hepatitis B surface antigen, and none had a history of excessive alcohol drinking or drug abuse. In control subjects, anti-HCV antibody test results were all negative and significant hepatic steatosis was not found by ultrasonography. The characteristics of patients and healthy subjects are summarized in Table 1.

**Table 1:** Patient and healthy subject characteristics.

Characteristics	Hepatitis C virus-related chronic liver disease	Healthy subjects	p-value
	(*n* = 69)	(*n* = 50)	
Age (years)	60.4±11.3	59.6±6.69	p = 0.35
Sex (M/F)	32/37	27/23	p = 0.41
BMI (Kg/m^2^)	23.2±3.27	22.4±2.59	p = 0.35
AST (IU/L)	52.0±30.3	23.1±5.27	*p<0.000000000001*
ALT (IU/L)	46.6±33.0	20.3±7.54	*p<0.00000001*
total CHO	156.2±36.9	213.0±28.8	*p<0.000000000001*
total TG	88.3±37.0	106.3±54.8	p = 0.08
Serotype, *n*			
Group 1	51		
Group 2	13		
Unclassified	5		
Fibrosis, *n*			
F 0	0		
F 1	17		
F 2	10		
F 3	20		
F 4	22		

p-values, when shown in italics such as *p<0.01*, indicate that there are significant differences. Values are shown as mean ± SD (standard deviation). BMI; Body Mass Index, AST; Aspartate transaminase, ALT; Alanine transaminase, CHO; cholesterol, TG; triglyceride.

### Description of Procedures or Investigations Undertaken

Liver biopsies were performed with a Tru-Cut needle (14G) under ultrasound guidance [9]. The biopsy specimens were reviewed by two experienced hepatologists. Fibrosis staging was evaluated based on the 5-point classification of Desmet et al. [10] as follows: F0 (no fibrosis), F1 (mild fibrosis), F2 (moderate fibrosis), F3 (severe fibrosis), and F4 (cirrhosis) (Table 1). In 90% of cases, the two hepatologists reached the same conclusion. When their results were discordant, a third hepatologist made the final determination.

At the time of the liver biopsy, venous blood was drawn after a 12-h overnight fast to determine blood chemistry values. Following routine laboratory tests, serum total TG and VLDL-TG levels were determined using residual sera. Serum total TG levels were measured by a conventional enzymatic assay (Auto L Mizuho TG-FR, Mizuho Medy, Tosu, Japan).

Serum VLDL-TG levels were determined using the surfactant-based homogenous assay proposed by Okada et al. [8]. In this assay protocol, two different nonionic surfactants, nonylphenol ethoxylate (Emulgen 911) and alkylpolyoxyethylene (Tergitol NP-10), were used to better differentiate VLDL and VLDL remnants (IDL) from other lipoproteins. HCV genotype was evaluated using the antibody serotyping method of Tsukiyama-Kohara et al. [11]–[13]. In this serotyping assay, HCV serotypes 1 and 2 corresponded to genotypes 1a/1b and 2a/2b, respectively, according to the classification of Simmonds et al. [14]. When the results of the serotyping assay were equivocal, PCR typing was conducted. Patients whose serotypes were not classified as serotype 1 or 2 were described as being of an unclassified type.

### Statistical Methods

Values are presented as mean ± SD (standard deviation). The results were analyzed using the Mann-Whitney *U*-test for comparisons between groups to determine their significance using the 4-Step Excel Statistics software application (OMS Publishing Inc., Tokorozawa, Japan, http://www.oms-publ.co.jp/index.html). Significance levels were set at p<0.05.

## Results

When comparisons were made between healthy subjects and patients with non-cirrhotic CH-C (stages F1–F3), total TG levels were similar (healthy subjects, 106.3±54.8 mg/dl; CH-C patients, 96.4±38.6 mg/dl; p = 0.61), but serum VLDL-TG levels were significantly lower (p = 0.044) in patients with non-cirrhotic CH-C (59.8±29.4 mg/dl) than in healthy subjects (78.5±48.9 mg/dl). In contrast, serum non-VLDL-TG levels were significantly higher (p<0.0001) in patients with non-cirrhotic CH-C (36.6±13.1 mg/dl) than in healthy subjects (27.8±10.6 mg/dl). The differences were more remarkable when serum VLDL-TG/non-VLDL-TG ratios were compared (healthy subjects, 2.87±1.56; CH-C patients, 1.65±0.68; p<0.000001).

**Figure 1:**
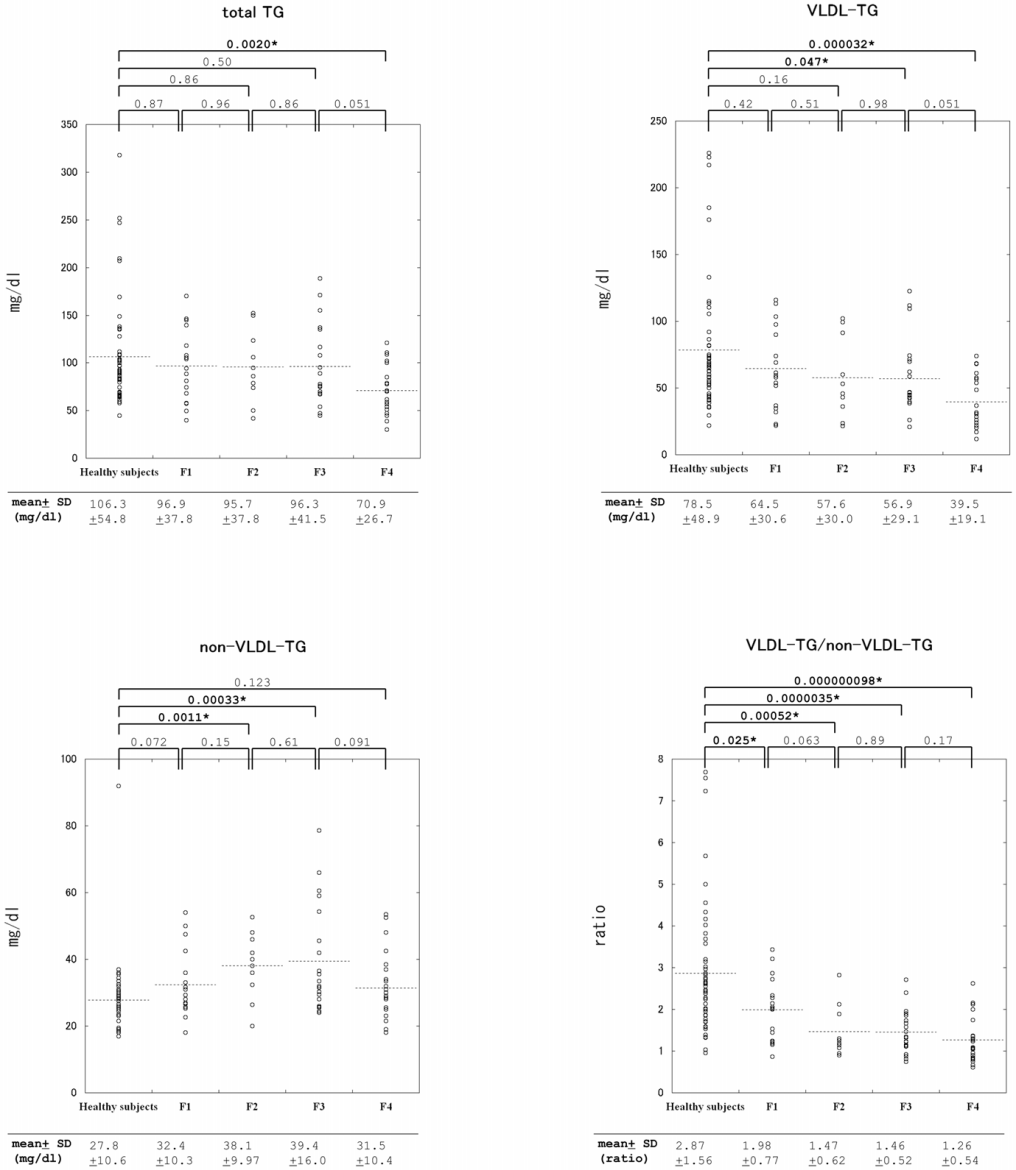
Scatter plots of serum total TG, VLDL-TG, non-VLDL-TG levels, and VLDL-TG/non-VLDL-TG ratios. P-values are indicated as the values between groups with bars. The mean value of each group is indicated at the bottom of each diagram. P-values, when indicated in bold with an asterisk (such as 0.025*), indicate significant differences. SD; standard deviation.

With regard to the disease stages classified with the 5-point Desmet scale, total TG levels in patients with CH-C did not demonstrate any significant differences at any stage compared with those in healthy subjects; however, a significant difference was observed only when the liver disease progressed to liver cirrhosis (stage F4) (Figure 1). In contrast, VLDL-TG levels were significantly different not only in cirrhosis (F4) but also in stage F3 of CH-C compared with those in healthy subjects (Figure 1).

Mean serum VLDL-TG levels demonstrated a tendency to decrease with progression of liver disease from F1 to F4 (Figure 1), while no significant differences were observed on comparing the healthy subjects and patients with stages F1, F1 and F2, F2 and F3, and F3 and F4 in the order of disease progression. In contrast, serum non-VLDL-TG levels were significantly increased in patients with stages F2 and F3 of CH-C compared to those in healthy subjects; however, no significant changes were observed in patients with liver cirrhosis (Figure 1).

As a result, serum VLDL-TG/non-VLDL-TG ratios were significantly decreased in both early and advanced stages of HCV-related chronic liver disease. Even in patients with the mildest form of CH-C (F1), serum VLDL-TG/non-VLDL-TG ratios alone were significantly different from those in healthy subjects (Figure 1).

In the present study, the changes in serum VLDL-TG and non-VLDL-TG levels were comparable between patients with HCV genotype 1 and HCV genotype 2.

## Discussion

The results of this study indicate that serum TG alterations, evaluated as decreases in the serum VLDL-TG/non-VLDL-TG ratio, occur as early as the stage F1 of CH-C. In contrast, no statistically significant differences were observed in serum total TG levels between healthy subjects and patients with stage F1 of CH-C as well as between healthy subjects and patients with non-cirrhotic CH-C (F1–F3).

Although it is known that the serum total TG level is decreased in advanced chronic liver disease [15], alterations that occur in TG metabolism in the early stages of HCV-related chronic liver disease (such as non-cirrhotic CH-C) remain to be elucidated. Because TGs in plasma are transported by a mixture of different lipoprotein species, selective measurement of these TG-rich lipoproteins will provide further insight into alterations of TG metabolism in liver injury. Because of the heterogeneity of the non-VLDL component, measurements of beta-lipoprotein levels will also be useful in assessing the stages of HCV-related chronic liver diseases [3].

Several possible mechanisms could lower the serum VLDL-TG/non-VLDL-TG ratio from the earliest stages of CH-C. Liver disease resulting from HCV infection might impair VLDL synthesis in hepatocytes. Previous studies with transgenic mice revealed that the HCV core protein inhibits microsomal transfer protein activity and causes impaired VLDL secretion [5], [6]. Indeed, mean serum VLDL-TG levels decreased with progression of liver disease from stages F1 to F4, and serum VLDL-TG levels were found to be significantly lower in patients with stage F3 of CH-C than those in healthy subjects (Figure 1).

The liver uptakes non-VLDL-TG in the form of free fatty acids and glycerols produced by peripheral lipases and hepatic triglyceride lipase [16] and in the form of lipoprotein particles including LDL [17]. Lipoprotein lipase, a peripheral lipase, preferentially hydrolyzes chylomicron-TG (CM-TG) and VLDL-TG [16]. The activity of hepatic triglyceride lipase, which preferably hydrolyzes HDL-TG [18], [19] that constitutes considerable portion of non-VLDL-TG [1], is known to be decreased in patients with parenchymal liver disease [20]–[22]. In addition, hepatic triglyceride lipase also serves as a ligand that facilitates uptake of lipoprotein particles including LDL [17]. Indeed, human subjects who have deficient activity of hepatic triglyceride lipase have increased plasma concentrations of HDL-TG and LDL-TG [23]–[25]. These factors may comprise mechanisms that explain why the serum non-VLDL-TG levels in stages F2 and F3 of CH-C were significantly increased (Figure 1). Thus, the decrease in serum VLDL-TG levels was attenuated by the increase in serum non-VLDL-TG levels, resulting in comparable serum total TG levels between healthy subjects and patients with non-cirrhotic CH-C. This means that serum total TG levels in patients with CH-C are dominated by two factors.

In view of the above findings, the premise that “it remains to be established whether or not the serum total TG levels in patients with CH-C are significantly lower than those in healthy subjects” is confirmed to have a logical basis. Furthermore, because of these changes in serum VLDL-TG and non-VLDL-TG levels, serum VLDL-TG/non-VLDL-TG ratios might indicate the clinical stage of HCV-related chronic liver disease more accurately than serum total TG or VLDL-TG levels alone.

When HCV-related liver disease progressed to stage F4, serum non-VLDL-TG levels, being different from those in patients with non-cirrhotic CH-C, did not demonstrate any significant difference when compared with those in healthy subjects (Figure 1). This may mean that the increase in serum non-VLDL-TG levels disappears when VLDL-TG secretion from the liver is considerably decreased because LDL-TG, that constitutes substantial portion of non-VLDL-TG, is generated from VLDL-TG released from the liver [16]. Not all non-VLDL-TG is generated from VLDL-TG. For example, CM is not generated from VLDL; however, CM-TG production is negligible in the fasting state [1]. This concept is supported by the fact that the mean serum VLDL-TG/non-VLDL-TG ratio in stage F4 CH-C (1.26±0.54) was lower than that in stage F3 (1.46±0.52); the p-value between patients with stage F4 and healthy subjects also was smaller than that between patients with stage F3 and healthy subjects (Figure 1).

The involvement of TG metabolism in CH-C pathogenesis has been established in experimental animals and cultured cells [5]–[7]. In contrast, conventional TG measurements are generally considered as poor indicators of CH-C progression until the end-stage of HCV-related chronic liver disease (cirrhosis). Our study has demonstrated that the measurement of the serum VLDL-TG/non-VLDL-TG ratio, which focuses on TG metabolic alterations during the development of HCV-related chronic liver disease, may be an early indicator of CH-C.

### Limitations

It should be noted that hepatic biopsy was not performed for the healthy subjects, and thus their livers were not confirmed to be completely normal. However, in control subjects, hepatitis B surface antigen and anti-HCV test results were all negative and their serum transaminase levels were within the reference intervals (Table 1). In control subjects, significant hepatic steatosis was not found by ultrasonography and none had a history of excessive alcohol drinking or drug abuse. The patients and healthy subjects in this study were from Japan, where HCV type 1 is the most common genotype [26]. Because genotype 3 is almost undetected in Japanese subjects, serum VLDL-TG/non-VLDL-TG ratios in genotype 3 patients remain to be tested.
